# Associations of sex hormone-binding globulin and testosterone with diabetes among men and women (the Saku Diabetes study): a case control study

**DOI:** 10.1186/1475-2840-11-130

**Published:** 2012-10-16

**Authors:** Atsushi Goto, Akemi Morita, Maki Goto, Satoshi Sasaki, Motohiko Miyachi, Naomi Aiba, Yasuo Terauchi, Mitsuhiko Noda, Shaw Watanabe

**Affiliations:** 1 Department of Diabetes Research, Diabetes Research Center, National Center for Global Health and Medicine, Tokyo, Japan; 2 National Institute of Health and Nutrition, Tokyo, Japan; 3 Department of Nutrition, College of Nutrition, Koshien University, Hyogo, Japan; 4 Department of Endocrinology and Metabolism, Yokohama City University Graduate School of Medicine, Kanagawa, Japan; 5 Department of Social and Preventive Epidemiology, School of Public Health, University of Tokyo, Tokyo, Japan; 6 Department of Nutrition and Life Science, Kanagawa Institute of Technology, Kanagawa, Japan; 7 Department of Diabetes and Metabolic Medicine, Center Hospital, National Center for Global Health and Medicine, Tokyo, Japan; 8 Department of Diabetes and Metabolic Medicine, Center Hospital/Department of Diabetes Research, Diabetes Research Center, National Center for Global Health and Medicine, 1-21-1 Toyama, Shinjuku-ku, Tokyo, 162-8655, Japan

**Keywords:** Sex hormone, Sex hormone-binding globulin, Sex difference, Fatty liver disease

## Abstract

**Background:**

Sex hormone-binding globulin (SHBG) levels and sex hormones have been implicated in the pathogenesis of type 2 diabetes and cardiovascular diseases. As fatty liver has been suggested to be a major determinant of SHBG levels, we examined whether the associations of SHBG and testosterone with diabetes were independent of fatty liver.

**Methods:**

We conducted a case–control study that included 300 diabetes cases (215 men and 85 women) and 300 matched controls from the Saku cohort study. Diabetes was defined by either fasting plasma glucose levels ≥126 mg/dL, 2-h post-load glucose levels ≥200 mg/dL after a 75 g oral glucose tolerance test, or diabetes diagnosed by physicians. We fitted conditional logistic regression models to examine the associations between SHBG and total testosterone levels with diabetes by sex. To evaluate the impact of fatty liver, we used the fatty liver index (FLI), a validated measure derived from serum triglyceride levels, body mass index (BMI), waist circumference, and γ-glutamyltransferase levels.

**Results:**

After adjusting for age, family history of diabetes, smoking, physical activity, BMI, and FLI, SHBG levels were inversely associated with diabetes among women (odds ratio [OR] comparing the highest with the lowest quartiles, 0.13 [95% confidence interval {CI}, 0.02–0.96]), but not among men. Similar patterns were observed in a subgroup analysis restricted to postmenopausal women"(OR, 0.12 [95% CI, 0.01–1.17]). In contrast, testosterone levels were inversely associated with diabetes among men (OR, 0.45 [95% CI, 0.23–0.89]), but not among women.

**Conclusions:**

Our findings suggest that SHBG in women and testosterone in men may be inversely associated with diabetes.

## Background

Emerging data indicate that sex hormone-binding globulin (SHBG) as well as adipose tissue function may play important roles in the development of type 2 diabetes [1-3] and cardiovascular diseases [4,5]. SHBG is synthesized primarily in the liver and binds to androgens and estrogens, thereby regulating the biologically active fraction of sex hormones [6]. Thus, the circulating SHBG level is a major determinant of the metabolic clearance of sex hormones, modulating the access of such sex hormones to their target tissues. The plasma membranes of various kinds of cells have been recently shown to be capable of binding specifically and with a high affinity to SHBG, mediating sex hormone signaling at the cell membrane through SHBG receptors [7,8].

Recent molecular epidemiologic studies mainly from Western populations have reported that genetically determined levels of SHBG were inversely associated with type 2 diabetes risk [2,9], lending support to the roles of SHBG in the development of type 2 diabetes. Importantly, fatty liver has been recently suggested to be a major determinant of decreased SHBG levels [10]. Because fatty liver is a strong risk factor of type 2 diabetes [11], investigating whether fatty liver accounts for the SHBG and type 2 diabetes relation may provide further insight into the role of SHBG in the pathogenesis of type 2 diabetes. However, the impact of liver function on the association has not been studied yet. Moreover, the previous studies examining the relation between SHBG and type 2 diabetes are mainly from European populations.

In addition, a sexually-dimorphic relationship between total testosterone levels and type 2 diabetes has been suggested. Studies in rodents reported that male androgen receptor knockout mice manifested late onset obesity with increased lipogenesis, insulin resistance, and impaired glucose tolerance [12-14], while obesity did not develop in the female androgen receptor knockout mice [14]. Further, a systematic review and meta-analysis reported that lower testosterone levels are associated with diabetes in men, whilst higher testosterone levels are associated with diabetes in women [1].

The purpose of this study was 1) to examine the associations of SHBG and testosterone with type 2 diabetes by sex among a Japanese population, independent of traditional diabetes risk factors, if such associations exist, 2) to evaluate whether the associations are independent of the fatty liver index (FLI), a validated measure of fatty liver, and 3) to examine whether sexual-dimorphism exists in the associations.

## Methods

### Study population

This was a cross-sectional analysis of the Saku cohort, which was launched from 2009 in Saku Central Hospital Human Dock Center in Saku city, Nagano Prefecture, Japan. Participants who visited for a health checkup between May 5, 2009 to September 30, 2010, and who agreed to participate in the cohort were included in the study. From the study population at baseline (*n* = 2,565), we excluded subjects with missing data (n = 30), age < 50 years old (*n* = 350), and age ≥ 80 (*n* = 16). Of the remaining 2,169 participants, 301 participants were defined to have diabetes. According to the WHO criteria [15], diabetes was defined by either fasting plasma glucose levels ≥126 mg/dL, 2-h post-load glucose levels ≥200 mg/dL after a 75 g oral glucose tolerance test, or diabetes diagnosed by physicians. Of the remaining 1,868 participants, 542 participants who were defined to have impaired glucose tolerance or impaired fasting glucose according to the WHO criteria were excluded. Control participants were randomly selected from the remaining 1,326 participants and individually matched to cases on age and sex (*n* = 301). Of these 602 participants, 1 case–control pair was excluded from the analysis because of no remaining serum sample for 1 male case, leaving 300 diabetes cases and 300 matched controls in the analysis. This study was reviewed and approved by the Ethical Committee of the National Institute of Health and Nutrition and Saku Central Hospital. Participants received a precise explanation of the study and provided their written informed consent.

### Anthropometric measurements

The height (cm) and weight (kg) of the subjects were measured with an automatic scale (Tanita, BF-220, Tokyo, Japan), in light clothing. The body mass index (BMI) was calculated as the weight (kg) divided by the squared height (m^2^). Waist circumference was measured twice at the umbilicus level while the subject was in a standing position using a fiberglass measuring tape; the average measurement was used for the analysis. Blood pressure was measured while the subject was in a sitting position using a validated automated blood pressure monitor (ES-H55; Terumo, Tokyo, Japan). The physical activity levels were obtained by asking the participants about their average frequency of physical activity: rarely/never, 1 to 3 times per month, 1 to 2 times per week, and more than 3 times per week.

### Laboratory procedures

Following an overnight fast, blood samples were collected at the time of each health checkup at the Saku Health Dock Center. Blood samples were collected in tubes containing EDTA and heparin for the measurement of the fasting plasma glucose, insulin, and HbA1c levels, and the remaining frozen serum samples were sent to the laboratory at the National Institute for Health and Nutrition and were stored in deep freezers. Serum gel separator tubes were used for the measurement of the total cholesterol, HDL cholesterol, and triglyceride (TG) levels. Routine laboratory blood analyses were performed at the Saku Central Hospital. HbA1c levels were measured using a high-performance liquid chromatography method (TOSOH HLC-723 G8; Tosoh Corporation, Tokyo, Japan), with intra- and inter-assay coefficients of variation (CVs) of 0.5–1.4% and 0.6–1.3%, respectively. The plasma glucose levels were analyzed using an enzymatic method (ECO glucose buffer; A&T Corporation, Kanagawa, Japan), with intra- and inter-assay CVs of 0.3–0.5% and 0.6–0.8%, respectively. The plasma insulin levels were analyzed using an electrochemiluminescence immunoassay (Modular E170; Roche Diagnostics, Mannheim, Germany), with intra- and inter-assay CVs of 0.5–2.0% and 3.2–3.6%, respectively. The serum albumin levels were measured with a modified bromocresol green method (Aqua-auto Kainos ALB Test Kit; KAINOS Laboratories Inc., Tokyo, Japan), intra- and inter-assay CVs of 1.2–2.2% and 1.7–2.4%, respectively. The serum γ-glutamyl-transferase (GGT) levels were analyzed with the Japan Society of Clinical Chemistry reference method (Cica Liquid γ-GT J; Kanto Chemical Co, Tokyo, Japan) and an autoanalyzer BM-2250 (Nihon Denshi, Tokyo, Japan), with intra- and inter-assay CVs of 1.0–3.7% and 0.96–3.65%, respectively. The serum total cholesterol, HDL cholesterol, and TG concentrations were determined using enzymatic methods (serum total cholesterol: Detaminar L TC II, Kyowa Medex, Tokyo, Japan; HDL cholesterol: Cholestest N HDL,Sekisui Medical Co. Ltd., Tokyo, Japan; and TG concentrations: Mizuho TG-FR Type II, Mizuho Medi, Saga, Japan) and an autoanalyzer BM-2250 (Nihon Denshi, Tokyo, Japan), with intra- and inter-assay CVs of ≤ 1.7% and ≤ 2.3%, respectively.

With the stored frozen samples, SHBG, testosterone, and estradiol were measured in the laboratory at SRL (Tokyo, Japan), blinded to cases and controls. Serum SHBG levels were analyzed with an immunoradiometric assay; (Siemens Medical Solutions Diagnostics, Los Angeles, CA, USA), and testosterone levels with an electro chemiluminescence immuno assay; (Roche Diagnostics GmbH, Mannheim, Germany). The intra- and inter-assay CVs for SHBG in the laboratory at SRL have been reported to be 1–3% and 7–8% [16], and the lower limit of detection for SHBG was 1.1 (nmol/L). The intra- and inter-assay coefficient of variations (CVs) were 1–3% and 2–3%, and the lower limit of detection was 0.04 ng/mL for testosterone. Of 600 total testosterone measurements, undetectable readings (1 control and 1 case among men, and 4 controls and 3 cases among women) were set to missing. Free testosterone levels were calculated using the methods described by Södergård et al. [17] and Vermuelen et al. [18].

The values for HbA1c were collected as Japan Diabetes Society (JDS) values, and then converted to National Glycohemoglobin Standardization Program (NGSP) values using the following conversion formula: HbA1c (NGSP) = 1.02×HbA1c (JDS)+0.25% [19].

We evaluated the fatty liver condition with the validated FLI derived from TG levels, BMI, waist circumference, and GGT levels as follows: exp[0.953×ln(TG) + 0.139×BMI + 0.718×ln(GGT) + 0.053×waist−15.745]/(1+exp[0.953×ln(TG) + 0.139×BMI + 0.718×ln(GGT) + 0.053×waist−15.745])×100 [20]. The FLI has a relatively high accuracy in detecting fatty liver (0.84 [95% CI, 0.81–0.87]) [20] and studies have shown that the FLI is associated with higher hepatic-related and cardiovascular disease mortality [21], incidence of diabetes [22], and insulin resistance, risk of coronary heart disease, and early atherosclerosis [23].

### Statistical analysis

We conducted all analyses by sex. Baseline characteristics were compared between case patients and control subjects using the paired *t*-test for continuous variables and the McNemar’s test for categorical variables. Pearson correlation coefficients (*r*) were calculated to evaluate associations between testosterone and SHBG levels, fasting insulin and glucose levels, HbA1c levels, BMI, and FLI among controls.

Odds ratios (ORs) and 95% confidence intervals were calculated according to quartiles based on the joint distribution of cases and controls by sex; the lowest quartile was used as the reference. We fitted conditional logistic regression models to estimate the association between SHBG and diabetes. In Model 1, we stratified on matched pairs using conditional logistic regression models. We further adjusted for smoking status (never, past, or current), physical activity (rarely/never, 1 to 3 times per month, 1 to 2 times per week, and more than 3 times per week), history of hypertension, family history of diabetes, alcohol use (almost none, occasional, or regular), menopausal status (women only), and BMI (continuous) (Model 2). To examine the impact of a fatty liver in the association, we additionally included the FLI (quartiles) in the model (Model 3). In Model 4, we further included levels of fasting insulin (quartiles), total testosterone (quartiles), and SHBG (quartiles) that were not examined as the primary independent variable. As sensitivity analyses, we additionally adjusted for total energy intake (quartiles) but this additional adjustment did not materially change the estimates. *P-*values for trend were computed based on median levels in categories. To assess nonmultiplicative interactions of testosterone and SHBG levels with gender, we fitted logistic models with product terms for each of these interactions, treating the biomarkers as continuous variables.

To further provide visual representation of the dose–response curve, we fitted restricted cubic spline models by including transformed variables of exposure variables to multiple logistic regression models (with 2 knots at the 33.3th and 67.7th percentiles) with adjustment for covariates included in Model 3 [24]. We conducted statistical analyses using SAS (version 9.3; SAS institute, Cary, NC) and STATA (version 12.0; StataCorp, College Station, TX).

## Results

The baseline characteristics of diabetes cases and matched-controls by sex are presented in Table 1. In both men and women, diabetes cases tended to have higher BMI, waist, HbA1c, fasting glucose levels, fasting insulin levels, and FLI than controls. Also, cases tended to have a history of hypertension and dyslipidemia, and a family history of diabetes. Diabetic men tended to have lower total testosterone levels than controls, while diabetic women tended to have higher C-reactive protein and lower SHBG levels than controls. In addition, 114 participants with diabetes (78 men and 36 women) were using anti-diabetic medications and 186 (137 men and 49 women) were not.

**Table 1:** Baseline characteristics between participants with cases of type 2 diabetes and control participants

**Characteristics**	**Cases**	**Controls**	*P*-values *
Men			
No.	215	215	
Age (yr)	64.5 ± 6.6	64.5 ± 6.6	
BMI (kg/m^2^)	24.1 ± 2.9	23.4 ± 2.7	0.01
Waist (cm)	87.0 ± 8.2	84.9 ± 7.7	0.006
Alcohol (% ≥ once/wk)	74.0	80.5	0.09
Smoking (% current)	19.5	15.3	0.25
Physical activity (% ≥ once/wk)	74.0	72.1	0.68
History of hypertension (%)	48.4	25.1	<0.001
History of dyslipidemia (%)	24.2	10.7	<0.001
Family history of diabetes (%)	29.8	13.0	<0.001
HbA1c (%)	6.6 ± 0.9	5.6 ± 0.3	<0.001
Fasting glucose (mg/dL)	128.5 ± 30.9	97.4 ± 6.2	<0.001
Fasting insulin (μIU/mL)	6.3 ± 5.4	4.5 ± 3.7	<0.001
Fatty liver index	38.1 ± 24.0	28.3 ± 19.6	<0.001
C-reactive protein (mg/dL)	0.23 ± 1.04	0.10 ± 0.17	0.07
Sex hormones			
SHBG (nmol/L)	72.6 ± 28.3	76.7 ± 28.9	0.12
Total testosterone (ng/mL)	4.7 ± 1.7	5.4 ± 1.7	<0.001
Free testosterone (ng/mL)	4.1 ± 1.6	4.5 ± 1.6	<0.002
Women			
No.	85	85	
Age (yr)	63.2 ± 7.0	63.2 ± 7.0	
BMI (kg/m^2^)	24.0 ± 3.7	21.5 ± 2.6	<0.001
Waist (cm)	85.6 ± 8.9	77.8 ± 8.6	<0.001
Alcohol (% ≥ once/wk)	24.7	20.0	0.45
Smoking (% current)	7.1	3.5	0.32
Physical activity (% ≥ once/wk)	74.1	70.6	0.60
History of hypertension (%)	57.7	21.2	<0.001
History of dyslipidemia (%)	35.3	15.3	0.002
Menopause (%)	82.4	72.9	0.13
Family history of diabetes (%)	41.2	17.7	0.002
HbA1c (%)	6.7 ± 1.1	5.7 ± 0.3	<0.001
Fasting glucose (mg/dL)	124.9 ± 31.5	96.0 ± 7.2	<0.001
Fasting insulin (μIU/mL)	6.8 ± 4.7	3.9 ± 2.0	<0.001
Fatty liver index	30.2 ± 23.3	13.6 ± 14.0	<0.001
C-reactive protein (mg/dL)	0.14 ± 0.22	0.06 ± 0.06	0.003
Sex hormones			
SHBG (nmol/L)	79.9 ± 35.9	102.7 ± 32.1	<0.001
Total testosterone (ng/mL)	0.18 ± 0.10	0.15 ± 0.07	0.06
Free testosterone (ng/mL)	0.13 ± 0.09	0.09 ± 0.05	<0.001

Abbreviations: SHBG, sex hormone-binding globulin; FLI, fatty liver index.

Data are means ± SD.

* Baseline characteristics were compared between case patients and controls using the paired *t*-test for continuous variables and McNemar’s test for categorical variables.

The correlation coefficients between testosterone, SHBG, BMI, FLI, and glycemia among control participants by sex are shown in Table 2. As a result of the free testosterone calculation from total testosterone, SHBG, and albumin levels by the formula of Södergård and Vermeulen et al. [17,18], free testosterone showed a strong positive correlation with total testosterone and a strong inverse correlation with SHBG in both men and women. In men, total testosterone levels were positively correlated with SHBG levels and inversely correlated with BMI, HbA1c, and FLI; free testosterone levels were inversely correlated with BMI and FLI. SHBG levels in men were inversely correlated with BMI and FLI, but not with glycemia. In women, total testosterone levels were not correlated with SHBG levels, BMI, fasting insulin, or glycemia, but showed a positive correlation with FLI; free testosterone levels were positively correlated with BMI, fasting insulin, glycemia, and FLI. SHBG levels in women were inversely correlated with BMI, HbA1c, fasting insulin, and FLI. 

**Table 2:** Correlation coefficients between sex hormones, SHBG, BMI, insulin, glucose, HbA1c, and FLI among control subjects by sex

**Men**	**Total T**	**Free T**	**SHBG**	**BMI**	**Fasting insulin**	**FPG**	**HbA1c**
Free T	0.48 (*P<*0.001)						
SHBG	0.61 (*P<*0.001)	−0.37 (*P<*0.001)					
BMI	−0.31 (*P<*0.001)	−0.15 (*P=*0.02)	−0.20 (*P<*0.001)				
Fasting Insulin	−0.03 (*P=*0.66)	−0.09 (*P=*0.17)	0.06 (*P=*0.37)	0.45 (*P<*0.001)			
FPG	−0.08 (*P=*0.22)	0.01 (*P=*0.84)	−0.08 (*P=*0.24)	0.06 (*P=*0.35)	0.17 (*P=*0.02)		
HbA1c	−0.15 (*P=*0.03)	−0.06 (*P=*0.34)	−0.05 (*P=*0.45)	−0.02 (*P=*0.77)	0.04 (*P=*0.59)	0.27 (*P<*0.001)	
FLI	−0.36 (*P<*0.001)	−0.14 (*P=*0.04)	−0.26 (*P<*0.001)	0.75 (*P<*0.001)	0.43 (*P<*0.001)	0.13 (*P=*0.05)	−0.06 (*P=*0.42)
Women	Total T	Free T	SHBG	BMI	Fasting insulin	FPG	HbA1c
Free T	0.81 (*P<*0.001)						
SHBG	0.06 (*P=*0.62)	−0.47 (*P<*0.001)					
BMI	0.16 (*P=*0.17)	0.41 (*P<*0.001)	−0.46 (*P<*0.001)				
Fasting Insulin	0.11 (*P=*0.33)	0.30 (*P=*0.01)	−0.34 (*P=*0.002)	0.53 (*P<*0.001)			
FPG	0.16 (*P=*0.16)	0.19 (*P=*0.09)	−0.16 (*P=*0.14)	0.14 (*P=*0.20)	0.45 (*P<*0.001)		
HbA1c	0.19 (*P=*0.08)	0.26 (*P=*0.02)	−0.25 (*P=*0.02)	0.13 (*P=*0.22)	0.26 (*P=*0.01)	0.46 (*P<*0.001)	
FLI	0.30 (*P=*0.01)	0.51 (*P<*0.001)	−0.38 (*P<*0.001)	0.77 (*P<*0.001)	0.62 (*P<*0.001)	0.35 (*P=*0.001)	0.30 (*P=*0.005)

Abbreviations: Total T, total testosterone; free T, free testosterone; SHBG, sex hormone-binding globulin; BMI, body mass index; FLI, fatty liver index.

**Figure 1:**
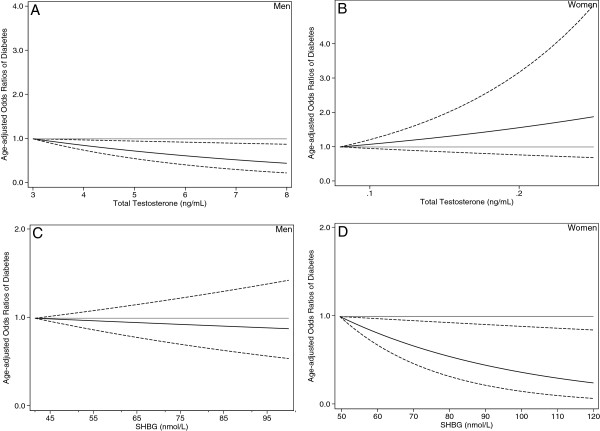
**The odds ratios of type 2 diabetes from restricted cubic spline models.** Abbreviation: SHBG, sex hormone binding globulin. Restricted cubic spline models with the inclusion of transformed variables in the conditional logistic regression models (with knots at the 33.3th and 67.7th percentiles) were used to estimate odds ratios (solid curve) with pointwise 95% confidence limits (dashed curves). The median value (Table 3) in the lowest quartile of each variable was used to estimate odds ratios. Adjustment was performed for matched strata, smoking status, physical activity, history of hypertension, family history of diabetes, alcohol use, menopausal status (women only), BMI, and the fatty liver index (quartiles). Panel **A** shows the results for total testosterone levels among men. Panel **B** shows the results for total testosterone levels among women. Panel **C** shows the results for SHBG levels among men. Panel **D** shows the results for SHBG levels among women.

After multivariable adjustment for age, family history of diabetes, smoking, physical activity, and BMI (Table 3; Model 2), SHBG levels were inversely associated with diabetes among women (OR comparing the highest with the lowest quartiles, 0.13 [95% CI, 0.02–0.86]), but not among men (OR, 0.73 [95% CI, 0.38–1.40]). Further adjustment for FLI slightly attenuated the association, but the strong inverse association observed in women remained (OR, 0.13 [95% CI, 0.02–0.96]) (Table 3; Model 3), suggesting that the inverse association of SHBG with diabetes is independent of fatty liver. Adjustment for TG, waist, and GGT in Model 3 instead of FLI did not materially alter the results. Spline curves clearly indicated that higher SHBG levels were markedly associated with a reduced chance of having diabetes in women, but not in men (Figure 1C and 1D). In addition, in a subgroup analysis restricted to postmenopausal women, similar patterns were observed. As compared with the lowest quartile of the intake, the adjusted ORs of diabetes across increasing quartiles were 1.00 (reference), 0.14 (95% CI, 0.01–1.48), 0.20 (95% CI, 0.04–1.07), and 0.12 (95% CI, 0.01–1.17) (*P*-value for trend = 0.07)among premenopausal women, however, we were not able to observe any pattern due to the small sample size (15 cases and 13 controls). *P*-value for interaction by sex was 0.007, indicating the importance of considering the sex-specific effects of SHBG. An additional adjustment for fasting insulin and total testosterone levels had little impact on the estimates (Table 3; Model 4).

**Table 3:** Odds ratio (95% CI) for type 2 diabetes according to quartiles of sex hormone and SHBG levels by sex

**Men**	**Quartiles**				
	**Q1 (lowest)**	**Q2**	**Q3**	**Q4 (highest)**	*P*for trend*
Testosterone (ng/mL)					
Median (range)	3.04 (0.06–3.84)	4.42 (3.85–4.88)	5.44 (4.90–6.20)	6.96 (6.21–12.80)	
(cases/controls)	69/39	54/55	49/56	43/64	
Model 1†	1.00	0.55 (0.31–0.98)	0.48 (0.27–0.84)	0.37 (0.21–0.66)	<0.001
Model 2‡	1.00	0.72 (0.38–1.37)	0.62 (0.32–1.20)	0.44 (0.23–0.84)	0.01
Model 3§	1.00	0.66 (0.34–1.29)	0.64 (0.33–1.26)	0.45 (0.23–0.89)	0.03
Model 4||	1.00	0.64 (0.32–1.30)	0.63 (0.29–1.34)	0.40 (0.18–0.88)	0.03
SHBG (nmol/L)					
Median (range)	42.1 (16.8–52.4)	62.2 (52.5–72.4)	81.8 (72.5–93.6)	112.0 (93.8–161.0)	
(cases/controls)	58/50	55/54	55/52	47/59	
Model 1†	1.00	0.87 (0.50–1.51)	0.87 (0.50–1.54)	0.67 (0.38–1.18)	0.17
Model 2‡	1.00	0.81 (0.43–1.51)	1.04 (0.55–2.00)	0.73 (0.38–1.40)	0.44
Model 3§	1.00	0.85 (0.44–1.64)	1.15 (0.59–2.24)	0.79 (0.41–1.53)	0.59
Model 4||	1.00	1.04 (0.52–2.10)	1.55 (0.73–3.27)	1.22 (0.56–2.67)	0.54
Women					
Testosterone (ng/mL)					
Median (range)	0.08 (0.04–0.10)	0.13 (0.11–0.15)	0.19 (0.16–0.22)	0.27 (0.23–0.48)	
(cases/controls)	20/24	19/22	22/21	21/14	
Model 1†	1.00	1.09 (0.49–2.47)	1.33 (0.55–3.17)	1.83 (0.76–4.40)	0.17
Model 2‡	1.00	0.76 (0.22–2.69)	1.05 (0.29–3.81)	1.90 (0.54–6.68)	0.27
Model 3§	1.00	0.72 (0.20–2.62)	0.97 (0.25–3.79)	2.02 (0.54–7.54)	0.25
Model 4||	1.00	0.74 (0.13–4.09)	2.82 (0.36–22.10)	9.20 (0.80–105.15)	0.046
SHBG (nmol/L)					
Median (range)	49.5 (12.9–60.8)	76.6 (61.3–89.4)	105.0 (89.5–124.0)	139.5 (125.0–163.0)	
(cases/controls)	33/10	20/22	20/23	12/30	
Model 1†	1.00	0.35 (0.13–0.91)	0.32 (0.13–0.78)	0.15 (0.05–0.40)	<0.001
Model 2‡	1.00	0.17(0.03–0.91)	0.19 (0.05–0.79)	0.12 (0.02–0.86)	0.03
Model 3§	1.00	0.18 (0.03–0.98)	0.20 (0.05–0.87)	0.13 (0.02–0.96)	0.048
Model 4||	1.00	0.21 (0.02–2.78)	0.23 (0.03–1.93)	0.03 (0.001–0.86)	0.06

Data are ORs (95% CI). Abbreviations: SHBG, sex hormone-binding globulin; ORs, odds ratios; CI, confidence interval * *P-*values for trend are based on median levels in categories. † Model 1: stratified on matched pairs using conditional logistic regression models. ‡ Model 2: further adjusted for smoking status, physical activity, history of hypertension, family history of diabetes, alcohol use, menopausal status (women only), and BMI. § Model 3: further adjusted for the fatty liver index (quartiles). || Model 4: further adjusted for levels of fasting insulin (quartiles), testosterone (quartiles), and SHBG (quartiles) that were not examined as the primary independent variable.

In contrast, after multivariable adjustment for known risk factors, total testosterone levels were inversely associated with diabetes among men (OR, 0.44 [95% CI, 0.23–0.84]), whereas a strong positive relation was suggested among women, although the estimates were very imprecise (Table 3; Model 2). Spline curves suggested a moderate inverse association between total testosterone and diabetes in men, but not in women (Figure 1A and 1B), suggesting that the total testosterone-diabetes relation observed among women (Table 3) was a statistically unstable estimate. The *P*-value for interaction by sex was 0.29, indicating insufficient evidence to support a sex-difference in the total testosterone and type 2 diabetes relation. Further adjustment for FLI (Table 3; Model 3), and SHBG and insulin levels (Table 3; Model 4) did not materially alter the estimates. In addition, we examined the relation between free testosterone levels and diabetes by sex. In a manner similar to total testosterone, after multivariable adjustment for known risk factors (Model 2), free testosterone levels were inversely associated with diabetes among men (OR, 0.44 [95% CI, 0.23–0.85]), but the estimates for women were very imprecise (OR, 15.28 [95% CI, 2.25–103.86]) (data not shown). The *P*-value for interaction by sex was 0.008. This suggests a sexual-dimorphism in the relation between free testosterone and diabetes; however, the effect measure modification might have been mainly driven by the SHBG-diabetes relation. Spline curves suggested a moderate inverse association between free testosterone and diabetes in men, but not in women (data not shown).

## Discussion

In this well-characterized case–control study in a Japanese population, we identified SHBG levels in women and total and free testosterone levels in men to be inversely associated with diabetes after multivariable adjustment for FLI and fasting insulin as well as known risk factors for diabetes. These findings suggest that SHBG in women and testosterone in men are not merely markers of liver function or hyperinsulinemia, adding support to the notion that SHBG and sex hormones may play important roles in the development of type 2 diabetes.

The findings for SHBG levels are in general agreement with previous findings. A systematic review and meta-analysis showed that SHBG was inversely associated with incident and prevalent diabetes in both men and women after adjustment for known risk factors for type 2 diabetes and the inverse association was stronger in women than in men [1]. In addition, previous studies in multi-ethnic populations have reported that the association remained consistent across ethnic groups: African-Americans, Hispanics, and Asians [25,26]. We did not observe the association in men and the interaction for gender was statistically significant (*P* = 0.007). Importantly, an earlier study in a largely Hispanic-American population from the San Antonio Heart Study found that the inverse association between SHBG and diabetes risk was strong in pre-menopausal women, weak in postmenopausal women, and negligible in men [27], suggesting a possible sexual-dimorphism in the association. Several studies examined whether the SHBG-diabetes relation was independent of sex hormones and reported that the relation remained [25,26].We observed that further adjustment for total testosterone levels had little impact on the estimates, suggesting that the mechanisms whereby SHBG contributes to diabetes development may be independent of testosterone [28]. Further, we investigated the impact of fatty liver on the SHBG-diabetes association with the FLI and found that further adjustment for FLI slightly attenuated the SHBG-diabetes association, but the association remained, indicating that SHBG may not merely be a marker of liver function, but may also be associated with diabetes, independent of fatty liver. Furthermore, recent genetic studies provided compelling evidence that genetically determined SHBG levels as well as variants of the *SHBG* gene are associated with type 2 diabetes risk [2]. Taken together, these findings support the role of SHBG in the development of diabetes.

Although the exact mechanisms by which SHBG influences the diabetes risk remain largely unclear, SHBG may contribute to the impairment of glucose metabolism through modulation of sex hormone bioavailability and direct activation of a specific receptor for SHBG [28]. Compensatory hyperinsulinemia resulting from insulin resistance has been proposed to be responsible for the apparent inverse SHBG-diabetes relationship [29]; however, it has been shown recently that a significant increase rather than decrease occurs in plasma SHBG levels after initiating insulin treatment in type 2 diabetic patients [30].

Total and free testosterone levels were inversely associated with diabetes among men, while a possible positive association was suggested among women after adjustment for known risk factors for type 2 diabetes in our study. These findings are in line with previous studies. In a systematic review and meta-analysis, Ding et al. found an inverse association between testosterone and diabetes in men; they found a positive association between testosterone and diabetes in women [1]. Growing evidence in human and rodents supports the roles of androgen in the glucose metabolism. Testosterone replacement resulted in improved glycemic control in hypogonadal men with type 2 diabetes [31] and androgen receptor knockout male mice manifested insulin resistance and impaired glucose tolerance [12]. Decreased testosterone levels may contribute to the development of diabetes by increasing insulin resistance via decreasing lean mass and increasing fat mass [32]. The inverse testosterone-diabetes relation in men remained after adjusting for BMI and fasting insulin in our study, suggesting that there may be an alternative pathway relating testosterone and diabetes.

Categorical analyses (Models 1–4) in our study suggested that, in contrast to men, total and free testosterone levels appeared to be positively associated with diabetes in women. The interaction for gender was significant for free testosterone (*P*-value = 0.008), although this might have been driven by the sex-difference in the SHBG-diabetes relation. However, the dose–response relation between testosterone levels and diabetes using cubic spline models indicated that no clear conclusions can be drawn regarding the testosterone-diabetes relation among women. Of importance, the total testosterone levels among women were far smaller than the levels among men; the median values of testosterone in the highest quartile of testosterone levels were 0.27 ng/mL among women and 6.96 ng/mL among men. This difference makes it difficult to directly compare the relation of testosterone with diabetes between men and women. Further experimental and observational studies are needed to better elucidate the sex-specific role of testosterone in the regulation of glucose metabolism.

Our study has several strengths. First, about 95% of the participants who did not self-report diabetes underwent the 75 g oral glucose tolerance test, which minimized the possibility of the inclusion of undiagnosed diabetic patients in the control group. Second, we obtained detailed measures of variables, allowing us to control for a number of potential confounding factors. Third, the control participants were randomly selected from the same population that gave rise to the cases which minimized the selection bias. Nevertheless, some limitations of the present study merit consideration. First, we were unable to establish a temporal relationship between the exposure (i.e., testosterone and SHBG) and diabetes. In addition, the observed associations may vary according to the duration of diabetes. Hyperinsulinemia or insulin resistance typically associated with diabetes might have resulted in decreased testosterone and SHBG levels (i.e., reverse causality). Further controlling for fasting insulin levels did not materially alter the results in our study, but use of sulphonylurea or insulin among participants with diabetes might have influenced the insulin levels. Therefore, the adjustment for fasting insulin levels may not have adequately captured the influence of hyperinsulinemia or insulin resistance. Second, we used the FLI, a validated surrogate maker for fatty liver, to examine the impact of fatty liver on the SHBG-diabetes association. Although this measure has been validated in independent studies, the FLI is not a perfect measure of fatty liver. Thus, residual confounding due to misclassification of fatty liver cannot be ruled out. Third, we did not ask women about the use of exogenous estrogen, which might have confounded the SHBG-diabetes association. The frequency of exogenous estrogen use in Japan is quite low (≈ 2.8%) [33], so such confounding would probably have little impact on our estimates. Finally, measurement errors for the exposures (i.e., testosterone and SHBG) might have biased the results. However, the errors are likely to be nondifferential with respect to the outcome, because case–control pairs were matched and laboratory personnel were blinded to case–control status. Therefore, such misclassifications would lead to an underestimation of the associations. Because most women had low testosterone values in our study and the immune assay in our study may be less sensitive than other assays, such as liquid chromatography–mass spectrometry, the possible inaccurate quantification at low values might have attenuated the association of testosterone with diabetes in women.

## Conclusions

Our findings suggest that SHBG in women and testosterone in men are associated with diabetes in a Japanese population. These findings lend support to the notion that SHBG and sex hormones may play important roles in the development of type 2 diabetes.

## Abbreviations

SHBG: Sex hormone-binding globulin; FLI: Fatty liver index; BMI: Body mass index; TG: Triglyceride; CV: Coefficient of variation; GGT: γ-glutamyl-transferase; IRMA: Immunoradiometric assay; JDS: Japan Diabetes Society; NGSP: National Glycohemoglobin Standardization Program; OR: Odds ratio.

## Competing interests

The authors declare that they have no competing interests.

## Authors’ contributions

AG wrote the manuscript and researched data. AM, MG, SS, NA, MM, YT, M.N., and SW contributed to the discussion and reviewed and edited the manuscript. All authors read and approved the final manuscript.
